# Comparison of the Level of Substance P and Neurokinin A in Gingival Crevicular Fluid of Sound and Symptomatic Carious Primary Teeth by ELISA

**Published:** 2017-07

**Authors:** Alireza Heidari, Mehdi Shahrabi, Marzieh Salehi Shahrabi, Mehdi Ghandehari, Pegah Rahbar

**Affiliations:** 1 Assistant Professor, Dental Research Center, Dentistry Research Institute, Tehran University of Medical Sciences, Tehran, Iran; Department of Pediatric Dentistry, School of Dentistry, Tehran University of Medical Sciences, Tehran, Iran; 2 Associate Professor, Department of Pediatric Dentistry, School of Dentistry, Tehran University of Medical Sciences, Tehran, Iran; 3 Postgraduate Student, Department of Pediatric Dentistry, School of Dentistry, Tehran University of Medical Sciences, Tehran, Iran; 4 Assistant Professor, Department of Pediatric Dentistry, School of Dentistry, Ahvaz Jundishapur University of Medical Sciences, Ahvaz, Iran

**Keywords:** Pulpitis, Neurokinin A, Tooth, Deciduous, Substance P

## Abstract

**Objectives:**

Pulpal inflammation is often associated with odontogenic pain. Dental pulp is abundantly innervated with sensory fibers encompassing neuropeptides. Neurokinin A (NKA) and substance P (SP) are important neuropeptides in the dental pulp that can cause neurogenic inflammation. Since no previous study has assessed dental pulp neuropeptides in children, this study aimed to compare the level of NKA and SP in gingival crevicular fluid (GCF) of sound and symptomatic carious primary teeth.

**Materials and Methods:**

Samples of GCF were obtained of 20 sound and 20 painful carious primary teeth. Enzyme-linked immunosorbent assay (ELISA) was used to quantify neuropeptides in GCF. Data were analyzed using paired t-test, ANOVA, Kolmogorov-Smirnov test and correlation coefficient test.

**Results:**

A significant difference was noted in the level of NKA in GCF of painful and sound teeth (2.23 pg/ml in painful, and 1.84 pg/ml in sound teeth, P<0.05). The difference between the two groups regarding SP was not significant (2.23 pg/ml in painful, and 2.02 pg/ml in sound teeth, P>0.05).

**Conclusions:**

The results showed that the level of NKA and SP was higher in GCF of painful teeth compared to that of sound teeth. This difference was statistically significant with regard to NKA. Thus, these neuropeptides can serve as indicators for pathological activities in teeth with symptomatic irreversible pulpitis.

## INTRODUCTION

Odontogenic pain is among the most common orofacial pains, often caused by pulpal inflammation. It has been stated that 48% of children with dental caries experience at least one episode of pain. A toothache negatively affects the quality of life and daily activities of children [1]. Stimulation of nociceptive neurons by physical stimuli or chemical mediators activates and sensitizes the nerves and creates pain signals, which are transferred to the trigeminal nucleus and induce the release of glutamate and neuropeptides such as substance P (SP). These molecules, by activating their receptors in post-synaptic neurons, transfer the pain signals to the thalamus, and from there to the brain cortex, where the odontogenic pain is perceived [2]. A wide range of inflammatory mediators is produced by different types of cells, which are responsible for tissue destruction [3]. Neuropeptides are one of the largest families of extracellular mediators. They are released from nerve fiber terminals following inflammatory stimulation and play a role in initiation and progression of inflammation [4, 5]. Evidence shows that cytokines, proteases, inflammatory mediators, growth factors and antimicrobial peptides are involved in pulpitis [6].

Neuropeptides also have an important role in neurogenic pulpitis and also in toothache mechanisms [2, 7, 8]. Dental pulp undergoes changes during neurogenic inflammation, as nerve fibers are sensitized and pain threshold decreases, while inflammatory response increases, and due to the release of vasoactive substances, plasma fluids and proteins exudate into the extracellular matrix and increase the pressure of the pulp at the site of injury [9].

Neurokinin A (NKA) and SP are neuropeptides from the family of tachykinins, which are accumulated in the secretory granules of unmyelinated sensory C-fibers. They are also present in primary sensory neurons and their peripheral nerve fibers [10]. They are released from the trigeminal nerve following physical and chemical stimulations, and cause vasodilation and increased pulpal pressure. These neuropeptides are the main neurotransmitters in generation and transfer of moderate to severe pain signals [11]. Evidence shows that pulpal pain increases calcitonin gene-related peptide (CGRP), NKA and SP [12, 13]. Even SP receptors increase during inflammation, as in irreversible pulpitis [14]. The interaction of dental pulp and periodontium has long been a topic of debate. The communication between the two has been shown with radiographic, histological and clinical evidence. The pulp and periodontium affect one another due to the existence of physiological and pathological communication pathways. The vascular system is the main, most recognized communication pathway between the dental pulp and periodontium. Considering the unique condition of the dental pulp and its direct communication with the periodontium via the apical foramen and accessory canals, it has been hypothesized that pathological processes in the dental pulp may cause changes in the gingival crevicular fluid (GCF) [15]. On the other hand, since sensory innervations of the dental pulp and periodontal tissue are derived from the same origin, stimulation of pulpal nerves may also stimulate the adjacent branches innervating the periodontal tissue [16, 17]. In recent decades, researchers have focused on analyzing the contents of GCF, aiming to find host markers for detection of diseases. Since the collection of GCF is easy and non-invasive, this method is extensively used [18].

Different methods such as radioimmunoassay, immunohistochemistry, and enzyme immunoassay have been used for measurement of the level of mediators; however, enzyme-linked immunosorbent assay (ELISA) is a more efficient technique for this purpose [16]. Considering the fact that previous studies have mainly focused on the presence of SP and NKA in the GCF of permanent teeth and not primary teeth, this study aimed to assess and compare the level of NKA and SP in GCF of sound and painful (carious) primary teeth. Assessment of the presence of these neuropeptides in GCF can help determine the pulpal status [16].

## MATERIALS AND METHODS

This analytical cross-sectional study was conducted on a group of pediatric patients presenting to the pediatric dentistry department of Tehran University of Medical Sciences in 2011. The study protocol was approved by the ethics committee of the university (16701-69-04-90). A total of 30 patients with irreversible pulpitis of a primary tooth (spontaneous, long-lasting and pulsating pain) and intact contralateral teeth were selected using targeted sampling. The inclusion criteria for the test group consisted of spontaneous pain in a primary tooth with irreversible pulpitis (diagnosed by clinical and radiographic examination), no previous history of pulp therapy and sound primary teeth in the contralateral quadrant. The exclusion criteria consisted of a history of taking antibiotics, anti-inflammatory drugs or analgesics in the past one month, history of systemic diseases affecting the periodontium, signs and symptoms of gingivitis, abscess, bleeding on probing, periapical or periodontal lesions on radiographs, and poor cooperation of the patient or parents not consenting to the study. After applying the inclusion and exclusion criteria, 20 patients (9 boys and 11 girls) were included in the study, and their first and last names, date of birth, gender, and position and type of teeth were recorded.

Demographic information of the patients including their first and last names, date of birth and gender as well as the tooth number, medical and dental history and history of a toothache were recorded after questioning the parents. In addition, the parents signed informed consent forms. New radiographs were obtained from the respective teeth. The control group included a sound primary tooth in the other quadrant of the same jaw in the same patient (no caries, asymptomatic, no periapical lesion). Level of pain experienced by the patients was recorded using a visual analog scale (VAS). The patients were asked to express their level of pain by picking a number between 0–10 [19].

### Preparation of samples:

To collect GCF samples, the area was isolated using cotton rolls and saliva ejector and was dried with gentle air spray for 15 seconds. GCF was collected using a #30 paper point (Aria Dent Co., Tehran, Iran), sterilized by gamma radiation. The paper strip was inserted by 1mm into the gingival crevice at the buccal surface until resistance was felt, and remained there for 30 seconds. Sampling was performed between 9–11 a.m. The paper strip was then placed in Eppendorf tubes containing 300μl of phosphate buffered saline (PBS, a nontoxic, isotonic and water-based solution with phosphate groups that maintain the pH at about 7.4), and was frozen at −20°C. The samples were sent to the immunology laboratory of Tehran University of Medical Sciences. The specimens were stored at −70°C until testing. ELISA was used to determine the concentration of NKA and SP in pg/ml. The tubes containing the samples of GCF of sound and carious teeth were coded in a blind fashion so that the technician was not aware of the contents of the tubes.

### ELISA:

The samples were kept at room temperature for one hour and were then centrifuged for 20 minutes. To assess the level of SP and NKA, EIA buffer, wash buffer and standard dilutions were prepared as recommended by the SP ELISA kit (Cayman Chemical, USA).

To prepare standard dilutions, 10 tubes were used; 900μl of the standard diluting solution was added to the first tube, and 500μl of this solution was added to the remaining tubes. 100μl of the standard concentrate was added to the first tube and was mixed using a shaker.

Next, 500μl of the contents of the first tube was transferred to the second tube, and this process was continued to the 10^th^ tube. Thus, 500, 250, 125, 62.5, 31.2, 15.6, 7.8, 3.9, 1.9 and 0.8 pg/ml dilutions were prepared. A single-well plate was allocated to each sample.

A single-well plate was also allocated per each standard dilution, and also to the solution with zero concentration. Next, 50μl of the solution in the 10^th^ tube, 50 μl of the respective sample and 50μl of SP EIA antiserum and SP AChE tracer were added to each single-well plate and were incubated overnight at 4°C. Unbounded proteins were rinsed the next day, and another bonding agent was used. This bonding agent was a molecule that could bind to the antibody and could also covalently bind to an enzyme like peroxidase. At the final step, a coloring solution was used, which could be converted to a colored product due to the action of the enzyme. The amount of antigen (SP) was measured by measuring the optical density by the dedicated software. To assess the concentration of NKA, standard dilutions were prepared as recommended by the NKA kit (Phoenix, USA). Five tubes were used; 900μl of the standard diluting solution was added to the first tube, and 500μl of this solution was added to the remaining tubes. 100μl of the standard concentrate was added to the first tube and was mixed using a shaker.

Next, 500μl of the contents of the first tube was transferred to the second tube, and this process was continued to the 5^th^ tube. Thus, 10, 5, 2.5, 1.2, and 0.6 pg/ml concentrations of the solution were obtained. Next, 50μl of the standard solution and 50μl of each sample were added to each single-well plate. By adding the sample and diluting solution to the wells containing an anti-NKA antibody, the NKA antigens present in the sample were binded to the antibody in the wells. Unbounded proteins were eliminated by rinsing with buffer.

The second anti-NKA antibody marked with horseradish peroxidase (HRP) enzyme was then added. Unbounded proteins were eliminated again by a rinse with buffer. Coloring substrate was then added and the optical density was read. The concentration of NKA in the sample was determined using a standard curve drawn by ELISA Reader (Model 3200, Dana). The association between color intensity and concentration of NKA was determined. Levels of NKA and SP in GCF of symptomatic and sound teeth were compared using paired sample t-test, while the correlation between levels of the two dependent variables were assessed by Pearson correlation coefficient. Statistical analysis was done using SPSS 22 for Windows (IBM Co., Chicago, IL, USA).

## RESULTS

This study was conducted on 20 patients (40 samples). 45% of the patients were males, and 55% were females, with a mean age of 6.80±1.47 years. Table 1 shows the demographic information of the participants. The level of pain was reported to be severe by all the patients using a VAS (≥5).

**Table 1.** Mean age (years) of male and female patients

**Sex**	**Mean**	**SD**	**N**
Male	6.66	1.22	9
Female	6.90	1.70	11
Total	6.80	1.47	20

SD=Standard Deviation

The mean concentration of SP inflammatory mediator in GCF of painful and sound primary teeth was found to be 2.23±0.69 and 2.02±0.37pg/ml, respectively. The mean concentration of NKA was 2.23±0.74 and 1.84±0.38 pg/ml, respectively. The results of Kolmogorov-Smirnov test showed that data regarding the concentration of NKA and SP mediators in GCF around painful and sound teeth had a normal distribution (P>0.05, Table 2).

**Table 2.** Results of Kolmogorov-Smirnov test with regard to the normality of NKA and SP levels in GCF of symptomatic and sound teeth

**Pain mediator**	**Z**	**P-value**
NKA (Symptomatic)	1.318	0.062
NKA (sound)	0.517	0.952
SP (Symptomatic)	0.783	0.571
SP (sound)	0.565	0.907

NKA=Neurokinin A, SP=Substance P

Paired t-test was then applied to compare the concentration of NKA and SP between the two groups of painful and sound primary teeth. No significant difference was noted in the concentration of SP between the two groups (P>0.05), but the difference between the two groups regarding NKA was statistically significant, and carious teeth had significantly higher level of NKA in their GCF than sound teeth (P<0.05, Table 3). Table 4 shows the correlation between the variables according to the correlation test.

**Table 3.** Results of paired t-test in comparing NKA and SP levels in GCF of symptomatic and sound teeth

Variable	Differences (Mean±SD)	P-value	95% Confidence interval	

			Upper bound	Lower bound
Level of NKA in symptomatic and sound primary teeth	0.39±0.68	0.019	0.71	0.073
Level of SP in symptomatic and sound primary teeth	0.203±0.82	0.282	0.59	−0.18

NKA=Neurokinin A, SP=Substance P, SD=Standard Deviation

**Table 4.** Results of correlation test between the levels of NKA and SP in GCF of symptomatic and sound teeth

**Quantitative variable**	**Correlation coefficient**	**P-value**
SP, NKA(symptomatic)	0.855	0.0001
SP, NKA(asymptomatic)	0.668	0.102

NKA=Neurokinin A, SP=Substance P

## DISCUSSION

The criteria currently used for assessment of pulp status are based on clinical and radiographic changes. Considering the limitations of conventional diagnostic methods, there is an obvious need for contemporary methods to detect changes. This study compared the level of NKA and SP in GCF of sound and painful carious primary teeth. To the best of the authors’ knowledge, this is the first study on the level of pain mediators in GCF of primary teeth, and there was no similar study to compare our results with. Therefore, we compared our findings with those of studies on permanent teeth. We found that the level of SP and NKA was higher in GCF around painful teeth, and this difference was statistically significant regarding NKA (2.23pg/ml in the test group, and 1.84pg/ml in the control group). However, the difference between the two groups was not significant regarding SP (2.23pg/ml in the test group, and 2.02pg/ml in the control group). We found that the levels of NKA and SP in painful teeth were significantly correlated, which is justifiable considering their similar origin and synergistic effects. In 2017, Heidari et al [20] conducted a similar study on permanent teeth and concluded that the level of NKA and SP in painful permanent teeth was higher than that in sound teeth, and this difference was significant with regards to both factors. However, in our study, the difference was significant only with regards to the level of NKA. This difference between the results of the two studies may be attributed to the difference between permanent and primary teeth. According to a study by Rodd and Boissonade [18] in 2003, the levels of SP and GRP in permanent teeth were higher than that in primary teeth. Awawdeh et al [11] in 2002 showed an increase in the level of NKA and SP in GCF of painful carious teeth compared to sound teeth. The difference in the levels of NKA and SP between their study and ours may be attributed to several factors such as the young age of the patients and evaluation of primary teeth in our study. In the mentioned study, all the subjects were over 18 years of age, and GCF was collected from painful teeth with irreversible pulpitis and sound contralateral teeth. Also, sampling duration was 30 seconds in our study, while Awawdeh et al reported sampling duration of one minute. Moreover, different paper strips were used in the two studies for GCF collection. We used #30 paper point, while Awawdeh et al used Perio papers, which may be more absorbent due to their larger surface. The difference in the concentrations may also be attributed to different laboratory procedures. In studies on coronal pulpal tissue, such as the studies by Rodd and Boissonade [21], Goodis et al [22] and Sattari et al [13], the level of SP in painful teeth was higher than that in asymptomatic sound teeth. Similarly, our study showed the higher level of SP in painful teeth compared to the controls; although this difference was not statistically significant, which may be due to smaller sample size in our study compared to the above-mentioned studies. In 2006, Caviedes-Bucheli et al [23] reported that the level of SP in painful teeth was significantly higher than that in the control group.

The difference between their study and ours is probably due to different methodologies since they evaluated pulpal samples of permanent teeth. Few studies have analyzed the contents of GCF and their association with pulpal conditions, pulpal pain and painful stimuli. In 2008, Avellan et al [24] showed that the level of SP in GCF increased following painful stimulation. However, the induced pain in their study was stimulatory and was therefore different from the pain of pulpal origin. This may explain the different reported values. In this study, the control teeth were sound teeth with no restorations, located in the other quadrant of the same jaw, corresponding to the teeth with pulpitis. In a systematic review conducted in 2015, Sakallioglu et al [5] reported that the level of CGRP, NKA, and SP was higher in restored teeth. In a review study in 2016, Rechenberg et al [6] concluded that the expression of inflammatory mediators in irreversible pulpitis is different from that in a healthy dental pulp. Their results were almost similar to ours; however, we only evaluated the level of NKA and SP. In a systematic review in 2017, Zanini et al [25] concluded that the levels of inflammatory mediators were different in teeth with reversible and irreversible pulpitis. They added that the level of tumor necrosis factor-alpha (TNF-α), receptor for advanced glycation end products (RAGE), matrix metallopeptidase-9 (MMP-9) and interleukin-8 (IL-8) increases following inflammation. Their results regarding the increase of the level of mediators in irreversible pulpitis were in agreement with ours. The difference between the two studies is probably due to different methodologies. We assessed the concentration of NKA and SP in GCF of sound teeth and those with irreversible pulpitis and noticed that these neuropeptides were present in GCF of both sound and painful carious teeth. Small sample size was a limitation of our study due to strict inclusion and exclusion criteria. Another limitation was the difficult detection of actual pain in children. Further studies are required to support the results regarding the efficacy of NKA and SP for detection of irreversible pulpitis.

## CONCLUSION

Based on the results of the present study, it may be concluded that pulpal inflammation increases the concentration of NKA and SP in GCF. Considering the role of these neuropeptides in vasodilation and release of other inflammatory mediators such as histamine, we may conclude that these two neuropeptides play a role in the development of dental pain and progression of inflammation. Considering the higher level of SP compared to NKA, it may be assumed that SP plays a more important role than NKA in this regard.
